# Outcomes of Adjuvant Oral versus Intravenous Fluoropyrimidines for High-Risk Stage II or Stage III Colon Adenocarcinoma: A Propensity Score-Matched, Nationwide, Population-Based Cohort Study

**DOI:** 10.7150/jca.42404

**Published:** 2020-04-12

**Authors:** Jiaqiang Zhang, Yu-Chun Yen, Lei Qin, Chia-Lun Chang, Kevin Sheng-Po Yuan, Alexander T.H. Wu, Szu-Yuan Wu

**Affiliations:** 1 Department of Anesthesiology and Perioperative Medicine, Henan Provincial People's Hospital, People's Hospital of Zhengzhou University, Zhengzhou, Henan, China.; 2 Biostatistics Center and School of Public Health, Taipei Medical University, Taiwan.; 3 School of Statistics, University of International Business and Economics, Beijing, China.; 4 Department of Hemato-Oncology, Wan Fang Hospital, Taipei Medical University, Taipei, Taiwan.; 5 Department of Otorhinolaryngology, Wan Fang Hospital, Taipei Medical University, Taipei, Taiwan.; 6 Ph.D. Program for Translational Medicine, Taipei Medical University, Taipei, Taiwan.; 7 Department of Food Nutrition and Health Biotechnology, College of Medical and Health Science, Asia University, Taichung, Taiwan.; 8 Division of Radiation Oncology, Lo-Hsu Medical Foundation, Lotung Poh-Ai Hospital, Yilan, Taiwan.; 9 Big Data Center, Lo-Hsu Medical Foundation, Lotung Poh-Ai Hospital, Yilan, Taiwan.; 10 Department of Healthcare Administration, College of Medical and Health Science, Asia University, Taichung, Taiwan.; 11 School of Dentistry, College of Oral Medicine, Taipei Medical University, Taipei, Taiwan.

**Keywords:** Colon adenocarcinoma, mortality, fluoropyrimidine, oral, intravenous.

## Abstract

**Objective**: We conducted this propensity score (PS)-matched, nationwide, population-based cohort study to estimate the effects of adjuvant oral or intravenous (IV) fluoropyrimidine in patients with high-risk stage II or III colon adenocarcinoma.

**Design**: Using PS matching, we minimized the confounding effects on adjuvant oral or IV fluoropyrimidine outcomes in patients with high-risk stage II or III resectable colon adenocarcinoma.

**Setting:** We selected patients from the Taiwan Cancer Registry database receiving adjuvant fluoropyrimidine monotherapy and divided them into those receiving IV fluoropyrimidine (IV group) and those receiving oral fluoropyrimidine (oral group).

**Results**: In both univariate and multivariate Cox regression analyses, the adjusted hazard ratio (aHR) derived for the oral group was 1.34 (95% CI: 1.19-1.51) compared with the IV group. Moreover, in both univariate and multivariate analyses, aHR derived for significant independent prognostic risk factors for poor overall survival were male sex, age ≥ 60 years old, pathologic stage III, right-sided colon cancer, low income, and high Charlson comorbidity index. However, intergroup differences were not significant among female patients or patients < 60 years old on multivariate analysis, including no difference in overall survival.

**Conclusions**: Adjuvant IV fluoropyrimidine is more suitable than adjuvant oral fluoropyrimidine for patients with stage II colon adenocarcinoma who have high-risk pathologic features or stage III colon adenocarcinoma.

## Introduction

Orally active fluoropyrimidines such as capecitabine and UFT offer increased convenience and the possibility of an improved therapeutic ratio, and adjuvant chemotherapy with oral fluoropyrimidines is at least as effective as that with intravenous (IV) fluoropyrimidines for stage III colon cancer in the UK 1. The benefits of oral fluoropyrimidine were addressed in a meta-analysis of Japanese trials involving 5 232 patients with resected stage I-III colon cancer who were randomly assigned to adjuvant oral fluoropyrimidine therapy or observation 2. Overall, oral fluoropyrimidine reduced the risk of death by 15%, but the absolute survival benefit for patients with stage III disease was only 2.5% 2.

Although recent trials suggest that the magnitude of benefit of oral fluoropyrimidine is at least equivalent to that of IV fluoropyrimidine 3, 4, these trials were performed in Western countries and Japan and had a relatively short follow-up time (≤5 years) 3-6. In addition, adjuvant fluoropyrimidine for colon cancer has been associated with more treatment-related toxicity in US patients taking oral fluoropyrimidine than in European and Japanese patients taking IV fluoropyrimidine 7. Tolerability to adjuvant oral fluoropyrimidine varies, with East Asian patients having the lowest and US patients the highest prevalence rates of adverse effects, dose reductions, and drug discontinuations 7. Moreover, oral fluoropyrimidine should be used cautiously in older patients with more treatment-related toxicity 7. However, data are inadequate for Chinese patients with resected stage II or III colon adenocarcinoma receiving adjuvant therapy. The Taiwanese government reports that 95%-97% of Taiwan's population is Han Cancer, which includes Hoklo, Hakka, and other ethnic groups originating from mainland China 8, 9. Considering this ethnic distribution, it is reasonable to extrapolate data of Taiwanese patients to Chinese patients with colon adenocarcinoma. It remains unclear whether adjuvant oral fluoropyrimidine has an equal survival effect to adjuvant IV fluoropyrimidine for Taiwanese patients with colon adenocarcinoma and whether sex, pathologic stage, age, or tumor location influences this effect.

In Taiwan, oral fluoropyrimidines are used as adjuvant therapy for stage II and III colon cancer. S-1 has not been approved for adjuvant therapy in stage II-III colon cancer by the Taiwan National Health Insurance Administration Ministry of Health and Welfare; it has been approved only for metastatic colon cancer when combined with IV oxaliplatin. Thus, in Taiwan, use of oral fluoropyrimidine has been relatively homogeneous for adjuvant therapy of stage II-III colon cancer. UFT and capecitabine are both orally bioavailable prodrugs that are converted to 5-fluorouracil (5-FU) through several enzymatic steps (acylamidase isoenzyme A, cytidine deaminase, and the cytochrome P(CYP)450 system in the liver), the last of which is mediated by thymidine phosphorylase (TP) in cancer cells 10, 11. In general, TP is overexpressed in colon cancer 12. which makes oral fluoropyrimidine chemotherapy suitable. Therefore, TP is a valid predictive biomarker for oral fluoropyrimidines 13. However, the liver enzymes or TP in patients with colon cancer might differ with respect to age, sex, and tumor stage or location 12-14. No study has investigated the differences in survival effects of adjuvant oral fluoropyrimidines with respect to age, sex, pathologic stage, and tumor location for high-risk stage II or III colon adenocarcinoma. Therefore, we conducted a propensity score (PS)-matched study to estimate the survival effects of adjuvant oral or IV fluoropyrimidine in patients with high-risk stage II and III colon adenocarcinoma after resection surgery.

## Patients and methods

### Data Source

The Taiwan Cancer Registry Database (TCRD) from the Collaboration Center of Health Information Application contains detailed cancer-related information 15-24.

### Study Cohort

From the TCRD, we enrolled patients who received a diagnosis of colon adenocarcinoma and underwent resection surgery between January 1, 2006, and December 31, 2014. The index date was the date of surgery. The follow-up duration was the index date to December 31, 2016. Our protocols were reviewed and approved by the Institutional Review Board of Taipei Medical University. The diagnoses of the enrolled patients were confirmed according to their pathological data, and patients who received a new diagnosis of colon adenocarcinoma and underwent surgery were confirmed to have no other cancer or metastasis. The inclusion criteria were a diagnosis of colon adenocarcinoma with an indication of surgery, age ≥ 20 years, Chinese ethnicity, and 7^th^ edition American Joint Committee on Cancer (AJCC) pathologic cancer stage IIB-IIIC without metastasis. The exclusion criteria were a history of cancer before the diagnosis of colon adenocarcinoma, unknown pathologic types, neuroendocrine neoplasms, hamartomas, mesenchymal tumors, lymphomas, signet ring cancers, mucinous carcinomas, missing sex data, unclear staging, and nonadenocarcinoma histology. Pathologic stage IIB-IIC for colon cancer included the following features: tumor invasion through the visceral peritoneum (pT4a), tumor invasion directly into or adherent to adjacent organs or structures (pT4b), presence of vascular, lymphatic, or perineural invasion 25, 26. In addition, we excluded patients with colon adenocarcinoma who received an insufficient number of cycles or duration of adjuvant chemotherapy (≥6-month duration) after surgery; had fewer than 12 lymph nodes examined; received prior chemotherapy, immunotherapy, or radiotherapy; or started adjuvant treatment after 8 weeks of surgery. All adjuvant treatments were initiated when no recurrence was recorded in the TCRD by well-trained Taiwan Cancer Registration professionals. Finally, we categorized the included patients into the 2 groups based on the type of adjuvant fluoropyrimidine received: those receiving IV fluoropyrimidine (IV group) and those receiving oral fluoropyrimidine (oral group). Adjuvant regimens of only fluoropyrimidine monotherapy were considered, and other adjuvant drugs (cetuximab, panitumumab, bevacizumab, oxaliplatin, and irinotecan) were not considered.

### Exposure Assessment

Comorbidities were scored using the Charlson comorbidity index (CCI) 27, 28. Only the comorbidities observed 6 months before the index date were included; comorbid conditions were identified according to the main International Classification of Diseases, Ninth Revision, Clinical Modification (ICD-9-CM) diagnosis codes for the first admission or ≥3 repeated main diagnosis codes for visits to an outpatient department.

To reduce the effects of potential confounding factors in intergroup comparisons, PS matching was employed. The PS was estimated using a multivariable logistic regression model, with treatment groups and potential confounding factors representing dependent variables and covariates, respectively. The following confounders were included in the PS matching: sex, age, pathologic stage, tumor location, income, and CCI. The protocols of IV or oral fluoropyrimidine chemotherapy are very different in cycles, which causes considerable variation in the total cumulative dose of fluoropyrimidine chemotherapy 5, 6, 14, 29, 30; thus, PS matching was not performed for these covariates. The details of duration, total dose, and fluoropyrimidine chemotherapy cycles are provided in Supplemental Table 1. All included patients were PS-matched at 1:1 by using the global optimum method 31.

### Endpoint

The endpoint was the all-cause mortality rate among the patients receiving adjuvant treatments, with the IV group as the control arm.

### Statistical Analysis

The Cox proportional hazards model with a robust variance estimator was also employed to calculate the hazard ratios (HRs) to determine whether factors such as different adjuvant oral or IV fluoropyrimidines chemotherapy, sex, age, pathologic stages, tumor locations, incomes, and CCIs were significant independent predictors (Table 2). The cumulative incidence of death was estimated using the Cox proportional hazards model with a robust variance estimator for overall survival (OS) in patients who received different adjuvant oral or IV chemotherapy and through stratified analysis of cancer stage, tumor location, sex, and age (Tables 3-6). After adjustment for confounders, the model was also used to characterize the time between the index date and all-cause mortality in the study patients. In the multivariate analysis, HRs were adjusted for sex, age, pathologic stage, tumor location, income, and CCI. All analyses were performed using SAS software (version 9.3; SAS, Cary, NC, USA). Two-tailed *P* < 0.05 was considered statistically significant. The cumulative incidence of death was estimated using the Kaplan-Meier method, and intergroup differences were determined using the log-rank test (Supplemental Figures 1-3).

## Results

After applying the exclusion criteria and PS matching algorithm, 4 936 patients with high-risk stage II and III colon adenocarcinoma were included, with 2 468 each in the oral and IV groups. The mean ages of the patients in the oral and IV groups were 59.9 and 58.4 years (Table 1), respectively, and the median follow-up durations were 5.8 and 8.1 years, respectively (Supplemental Table 1). The 10-year interval of age was almost balanced for the 2 groups, as were the CCI scores (Table 1). The AJCC pathologic stages were similar in the 2 groups. Sex, tumor location, income, and CCI were similar between the groups after PS matching, and the standardized mean differences (SMDs) were all <0.1 (Table 1) 32. The 5-year number of deaths for adjuvant IV form chemotherapy group is 837, for adjuvant oral form chemotherapy group is 1367. The number of deaths overall is 2204. The follow-up durations were not matched because the survival time was inconsistent in the treatment groups (Supplemental Table 1).

Multivariate Cox regression analysis revealed that adjuvant oral or IV fluoropyrimidines were significant independent predictors of OS (Table 2). Both univariate and multivariate analyses indicated that adjuvant IV fluoropyrimidine was a significant independent prognostic factor for a relatively higher OS, with the adjusted HR (aHR) for the oral group being 1.34 (95% CI: 1.19-1.51) compared with the IV group (Table 2). Moreover, in both univariate and multivariate analyses, the significant independent prognostic risk factors for poor OS were the following: male sex, age ≥ 60 years, pathologic stage III, right-sided colon cancer, low income, and high CCI (Table 2).

The cumulative incidence of death was estimated using the Cox proportional hazards model with PS matching and a robust variance estimator in the oral and IV groups and stratified by pathologic stage, tumor location, sex, and age. The results indicated that IV fluoropyrimidine is better than oral fluoropyrimidine as adjuvant chemotherapy irrespective of pathologic stage and tumor location (Tables 3 and 6). However, for female patients or patients < 60 years old, no significant difference was observed between the 2 groups (Tables 4 and 5).

The OS estimates obtained using the Kaplan-Meier method were employed to analyze the risk of death associated with the adjuvant oral or IV regimens (Supplemental Figures 1-3). The 5-year OS rates were 83.44% and 76.75% in the IV and oral groups, respectively (log-rank *P* < 0.0001; Supplemental Figure 1). The survival rate of patients with high-risk stage II colon adenocarcinoma in the IV group was superior to that in the oral group (log-rank *P* = 0.0602; Supplemental Figure 2). The 5-year OS rates at high-risk stage II were 90.01% and 84.35% in the IV and oral groups, respectively (Supplemental Figure 2). The survival rate of patients with stage III colon adenocarcinoma in the IV group was superior to that in the oral group (log-rank *P* < 0.0001; Supplemental Figure 3). The 5-year OS rates at stage III were 77.35% and 69.88% in the IV and oral groups, respectively (Supplemental Figure 3). According to our data and sample size with significance level of 0.05, the power of comparing adjuvant IV form chemotherapy group and adjuvant oral form chemotherapy group is 94%. If we consider the multiple testing and change the significance level to 0.0053, the power of comparing adjuvant IV form chemotherapy group and adjuvant oral form chemotherapy group is 99%. Therefore, the power is available given the current sample size.

## Discussion

Our PS matching was well performed, and the SMD was <0.1, indicating good matching for all covariates. Both univariate and multivariate Cox regression analyses revealed the following significant independent prognostic risk factors for poor OS: oral fluoropyrimidine, male sex, age ≥ 60 years, pathologic stage III, right-sided colon cancer, low income, and high CCI. To clarify the survival effects of oral or IV fluoropyrimidine in resected stage II-III colon adenocarcinoma, we used a stratified Cox proportional hazard regression model for sex, age, pathologic stage, and tumor location according to the outcomes in Table 2. We discovered that oral fluoropyrimidine had a higher risk of all-cause mortality compared with IV fluoropyrimidine in Chinese patients with resected stage II-III colon adenocarcinoma. However, survival effects were similar in female and young (<60 years) Chinese patients irrespective the mode of administration of adjuvant fluoropyrimidine.

Oral capecitabine is a prodrug of 5-FU; it is absorbed intact from the intestine 33 and converted into doxifluridine by the sequential actions of acylamidase isoenzyme A and cytidine deaminase in the liver 34. Women were reported to have elevated maximum 5-FU concentration (Cmax) and reduction in clearance after oral capecitabine as well as a greater area under the curve (AUC) compared with men 14. Thus, there are differences in the pharmacokinetics of capecitabine and its metabolites between male and female patients, and cytidine deaminase activity may be higher in females 14. This outcome might explain why female patients with resected stage II-III colon adenocarcinoma using oral capecitabine had comparable survival with those receiving IV fluoropyrimidine. In addition, UFT is a combination of tegafur, a prodrug of 5-FU, and uracil in a molar ratio of 1:4 35. Tegafur is converted into 5-FU by the hepatic cytochrome P450 pathway 36, whereas uracil enhances the half-life of the converted 5-FU by competing for its degradation by dihydropyrimidine dehydrogenase (DPD), which is the rate-limiting enzyme in 5-FU catabolism 37. The hepatic cytochrome P450 pathway has higher activity in women than in men 38, 39, which leads to a higher conversion rate of 5-FU in women. Sex-based differences in drug metabolism are the primary cause of sex-dependent pharmacokinetics and reflect underlying sex differences in the expression of hepatic enzymes relevant to drug metabolism, including cytochromes P(CYP)450 38, 39. Sex differences in drug metabolism and pharmacokinetics are due in part to the female-predominant expression of CYP3A4, the most critical P450 catalyst of drug metabolism in the human liver 38, 39. Moreover, higher DPD activity is evident in South-West Asian male subjects 40. These reasons might explain why survival was similar for oral UFT and IV fluoropyrimidine treatment among female patients; 5-FU is degraded relatively easily in men 33, 34, and the higher 5-FU activity in women than in men leads to the eradication of more cancer cells (Table 5).

In elderly patients, a 3-fold higher Cmax and 2-fold higher AUC of capecitabine were observed, which corresponded to lower capecitabine clearance compared with control patients (<60 years old) 14. The higher Cmax and AUC of the prodrug capecitabine in elderly patients leads to insufficient Cmax and AUC of 5-FU; this explains why the response rates to 5-FU in elderly patients with colon cancer who were taking oral prodrugs of 5-FU were usually lower than those in the general population 14, 33, 41. These findings might explain why the survival in young patients with resected stage II-III colon adenocarcinoma taking oral fluoropyrimidine was comparable to the survival of those taking IV fluoropyrimidine in our study. In addition, CYP450 activity is higher in young patients than in older patients 42, 43, leading to higher conversion rates of the prodrug to 5-FU. Age-based differences in drug metabolism are the primary cause of age-dependent pharmacokinetics and reflect underlying age differences in the expression of hepatic enzymes active in the metabolism of drugs, including CYP450 43, 44. Moreover, higher DPD activity was evident in elderly patients, leading to enhanced degradation of 5-FU 45; this might be why young patients had similar survival regardless of whether they received oral UFT or IV fluoropyrimidine. The higher 5-FU activity in young patients causes more toxic effects in cancer cells.

We found that IV fluoropyrimidine had superior OS compared with oral fluoropyrimidine in patients with resected stage II or III colon adenocarcinoma irrespective of the stage of colon adenocarcinoma. In the multicenter international study of oxaliplatin, fluoropyrimidine, and leucovorin in the adjuvant treatment of colon cancer trial, the addition of oxaliplatin to fluoropyrimidine could overcome the chemoresistance of colon cancer with deficient mismatch repair, especially in high-risk stage II or stage III colon cancer 46, 47. In our study, IV fluoropyrimidine decreased the all-cause mortality rate compared with oral fluoropyrimidine. Considering this and our outcomes together, we suggest combining oxaliplatin with IV fluoropyrimidine.

Studies have reported conflicting results regarding whether different adjuvant chemotherapy regimens exert different effects on left- and right-sided colon cancers because of differences in gene mutation 48-50. Tumor location might be a proxy for molecular biology. For example, mutations in BRAF or KRAS were reported to be more common in right-sided colon cancers, whereas left-sided colon cancers were more likely to be nonmutated 49. Data from The Cancer Genome Atlas have indicated a difference in the distribution of the consensus molecular subtypes between right- and left-sided colon cancers 51. In a meta-analysis of 66 studies including 1 427 846 patients with all stages of the disease, left-sided primary tumor location was associated with a significantly lower risk of death, independent of cancer stage, race, use of adjuvant chemotherapy, study publication year, and study quality 52. Consistent with this, our results showed that right-sided colon adenocarcinoma had higher all-cause mortality compared with left-sided colon adenocarcinoma. Additionally, adjuvant IV fluoropyrimidine demonstrated superior survival to oral fluoropyrimidine in resected colon adenocarcinoma, irrespective of tumor location. Thus, our findings indicated that IV fluoropyrimidine is essential for colon adenocarcinoma at any location, irrespective of the distribution of the consensus molecular subtypes.

A strength of our study is that it was a large cohort study that involved PS matching designed to determine whether adjuvant IV or oral fluoropyrimidine had better OS for Chinese patients with high-risk stage II or stage III colon adenocarcinoma. Our study is also the first to evaluate the changes in survival effects with respect to age, sex, pathologic stage, and tumor location in patients with resected colon adenocarcinoma taking adjuvant oral fluoropyrimidine compared with those taking IV fluoropyrimidine. Our findings reveal that adjuvant IV fluoropyrimidine might result in superior OS in male or elderly patients with high-risk stage II and stage III colon adenocarcinoma compared with adjuvant oral fluoropyrimidine use. However, female or young (<60 years old) cancer patients with colon adenocarcinoma could take either IV or oral fluoropyrimidine because the 2 routes of administration exhibited similar all-cause mortality in these subgroups. In other cancer patients with stage II or stage III colon adenocarcinoma, adjuvant IV fluoropyrimidine is recommended. All , covariates, IV or oral fluoropyrimidine forms, and pathologic stages in our study were more homogeneous than those in other studies. The outcomes in our study may be a useful reference for future clinical trials or clinical practice.

This study has some limitations. First, we did not determine the toxicity induced by the adjuvant oral or IV fluoropyrimidine, which could have introduced bias in treatment-related mortality estimates. Moreover, studies have demonstrated some differences in complications and toxicity between adjuvant oral and IV fluoropyrimidines treatment among different regions, such as North America, Europe, and Japan 2-5, 7. However, the study has shown more treatment-related toxicity in oral fluoropyrimidines 7. Thus, we believe that the survival benefits associated with IV fluoropyrimidine may be underestimated. Second, because all enrolled patients were of Chinese ethnicity, our results should be cautiously extrapolated to non-Chinese patients. Third, we did not have molecular data for patients with colon adenocarcinoma. However, despite encouraging preliminary data linking molecular findings to prognosis and potentially better prognostic stratification relative to tumor-node- metastasis staging alone, no single molecular marker, multiple marker profile, or gene expression panel has emerged as having predictive utility 53-63. Fourth, the diagnoses of all comorbid conditions were based on ICD-9-CM codes. Nevertheless, the Taiwan Cancer Registry Administration randomly reviews charts and interviews patients to verify the accuracy of the diagnoses, and hospitals with outlier chargers or practices may be audited and subsequently be heavily penalized if malpractice or discrepancies are identified. Fifth, compliance to adjuvant oral or IV fluoropyrimidine was not determined. However, our data indicated that in female and young patients, survival effects did not differ between the oral and IV groups. No study has reported better compliance of drugs in female or young patients with colon cancer. Thus, our outcomes are more in line with real-world results. To obtain crucial information on population specificity and disease occurrence, a large-scale randomized trial comparing carefully selected patients undergoing suitable treatments is essential. Finally, the TCRD does not contain information regarding dietary habits, socioeconomic status, or body mass index, all of which may be risk factors for mortality. However, considering the magnitude and statistical significance of the observed effects in this study, these limitations are unlikely to affect the conclusions.

## Conclusions

Adjuvant IV fluoropyrimidine is more suitable than adjuvant oral fluoropyrimidine for patients with stage II colon adenocarcinoma who have high-risk pathologic features or stage III colon adenocarcinoma. There is no OS difference between adjuvant IV and oral fluoropyrimidine treatment in female or young patients.

## Supplementary Material

Supplementary figures and tables.

## Figures and Tables

**Table 1:** Characteristics of adjuvant oral or intravenous fluoropyrimidine monotherapy in patients with colon adenocarcinoma who underwent resection and their propensity score-matched cohort.

	Oral Fluoropyrimidine N = 2,468	IV Fluoropyrimidine N = 2,468	SMD
Sex			
Male	1365.0 (55.3)	1365.0 (55.3)	0.0000
Female	1103.0 (44.7)	1103.0 (44.7)	0.0000
Age, mean (SD)	59.9 (12.5)	58.4 (13.3)	0.0584
<40	130 (5.3)	137 (5.6)	0.0140
40-49	282 (11.4)	300 (12.2)	0.0238
50-59	576 (23.3)	602 (24.4)	0.0257
60-69	696 (28.2)	747 (30.3)	0.0477
70-79	694 (28.1)	592 (24.0)	0.0949
≥80	90 (3.6)	90 (3.6)	0.0000
AJCC pathological stage			
High-risk stage IIB-IIC	1285 (52.1)	1285 (52.1)	0
III	1183 (47.9)	1183 (47.9)	0
Tumor location			
Left	1320 (53.5)	1233 (50.0)	0.0705
Transverse	61 (2.5)	54 (2.2)	0.0193
Right	1087 (44.0)	1181 (47.9)	0.0762
CCI			
0	374 (15.2)	368 (14.9)	0.0070
1	477 (19.3)	429 (17.4)	0.0519
2	334 (13.5)	306 (12.4)	0.0333
3	199 (8.1)	166 (6.7)	0.0481
≥4	1084 (43.9)	1199 (48.6)	0.0940
CCI score			
Mean (SD)	4 (3.5)	4.2 (3.4)	0.066
Median (IQR)	3 (6.0)	3.0 (6.0)	-
Income			
≤19,047	645 (26.1)	668 (27.1)	0.0050
19 048-21 000	748 (30.3)	729 (29.5)	0.0309
21 001-36 300	605 (24.5)	606 (24.6)	0.0123
>36 300	470 (19.0)	465 (18.8)	0.0161

SMD, standardized mean difference; CCI, Charlson comorbidity index; SD, standard deviation; IQR, interquartile range; AJCC, American Joint Committee on Cancer; IV, intravenous. Values are presented as number (%) unless otherwise indicated. test: Compare the mean among the 2 treatment groups. Mann-Whitney U test: Compare the median among the 2 treatment groups. Chi-square test: Examine the relationships between treatment groups and categorical factors, such as sex, age group, stages, tumor locations and CCI groups.

**Table 2:** Cox proportional hazards regression model with a robust variance estimator for evaluating the risk of death among patients with colon adenocarcinoma who received adjuvant oral or intravenous fluoropyrimidine chemotherapy.

		Univariate analysis				Multivariate analysis*	
	HR	95% CI	*P* value		aHR	95% CI	*P* value
Adjuvant chemotherapy				Adjuvant chemotherapy			
IV fluoropyrimidine	1.00			IV fluoropyrimidine	1.00		
Oral fluoropyrimidine	1.35	1.21-1.50	<.0001	Oral fluoropyrimidine	1.34	1.19-1.51	<.0001
Age							
<40	1.00			<40	1.00		
40-49	0.83	0.52-1.33	.4379	40-49	0.89	0.56-1.42	.6183
50-59	1.19	0.78-1.80	.4277	50-59	1.09	0.72-1.66	.6849
60-69	2.22	1.49-3.32	<.0001	60-69	1.74	1.16-2.60	.0076
70-79	4.12	2.77-6.12	<.0001	70-79	2.81	1.88-4.20	<.0001
≥80	8.12	5.29-12.46	<.0001	≥80	5.04	3.26-7.81	<.0001
Sex							
Female	1.00			Female	1.00		
Male	1.38	1.22-1.56	<.0001	Male	1.33	1.18-1.50	<.0001
AJCC pathologic stages							
High-risk stage IIB-IIC	1.00			High-risk stage IIB-IIC	1.00		
III	2.60	2.28-2.95	<.0001	III	1.60	1.37-1.85	<.0001
Tumor location							
Left	1.00			Left	1.00		
Transverse	1.16	0.81-1.66	.4168	Transverse	1.25	0.87-1.81	.2271
Right	1.09	0.97-1.22	.1613	Right	1.19	1.05-1.34	.0054
CCI				CCI			
0	1.00			0	1.00		
1	1.55	1.17-2.06	.0023	1	1.35	1.02-1.80	.036
2	1.85	1.38-2.47	<.0001	2	1.35	1.01-1.81	.044
3	2.41	1.77-3.30	<.0001	3	1.58	1.15-2.16	.0044
≥4	3.83	3.03-4.85	<.0001	≥4	2.02	1.56-2.60	<.0001
Income				CCI			
≤19,047	1.00			0	1.00		
19 048-21 000	0.84	0.73-0.97	.0201	1	0.91	0.78-1.05	0.2004
21 001-36 300	0.61	0.51-0.72	<.0001	2	0.83	0.70-0.99	0.0352
>36 300	0.54	0.46-0.64	<.0001	3	0.68	0.57-0.81	<.0001

*All aforementioned variables were used in the multivariate analysis. CCI, Charlson comorbidity index; AJCC, American Joint Committee on Cancer; IV, intravenous; aHR, adjusted hazard ratio.

**Table 3:** AJCC stage-stratified Cox proportional hazards regression model with a robust variance estimator for evaluating the risk of death among patients with colon adenocarcinoma who received adjuvant oral or intravenous fluoropyrimidine chemotherapy.

High-risk stage IIB-IIC				Stage III			
Adjuvant Treatment	aHR^*^	95% CI	*P* value	Adjuvant Treatment	aHR^*^	95% CI	*P* value
IV fluoropyrimidines	1.00			IV fluoropyrimidines	1.00		
Oral fluoropyrimidines	1.29	1.04-1.60	.0198	Oral fluoropyrimidines	1.37	1.19-1.57	<.0001

*All the aforementioned variables in Table 2 were used in the multivariate analysis. aHR, adjusted hazard ratio.

**Table 4:** Age-stratified Cox proportional hazards regression model with a robust variance estimator for evaluating the risk of death among patients with colon adenocarcinoma who received adjuvant oral or intravenous fluoropyrimidine chemotherapy.

≤60 years old				>60 years old			
Adjuvant Treatment	aHR^*^	95% CI	*P* value	Adjuvant Treatment	aHR^*^	95% CI	*P* value
IV fluoropyrimidines	1.00			IV fluoropyrimidines	1.00		
Oral fluoropyrimidines	1.06	0.83-1.37	.6358	Oral fluoropyrimidines	1.45	1.28-1.65	<.0001

*All the aforementioned variables in Table 2 were used in the multivariate analysis. aHR, adjusted hazard ratio.

**Table 5:** Sex-stratified Cox proportional hazards regression model with a robust variance estimator for evaluating the risk of death among patients with colon adenocarcinoma who received adjuvant oral or intravenous fluoropyrimidine chemotherapy.

Male				Female			
Adjuvant Treatment	aHR^*^	95%CI	*P* value	Adjuvant Treatment	aHR^*^	95%CI	*P* value
IV fluoropyrimidines	1.00			IV fluoropyrimidines	1.00		
Oral fluoropyrimidines	1.44	1.24-1.68	<.0001	Oral fluoropyrimidines	1.18	0.97-1.42	.0933

*All the aforementioned variables in Table 2 were used in the multivariate analysis. aHR, adjusted hazard ratio.

**Table 6:** Tumor location-stratified Cox proportional hazards regression model with a robust variance estimator for evaluating the risk of death among patients with colon adenocarcinoma who received adjuvant oral or intravenous fluoropyrimidine chemotherapy.

Left				Transverse				Right			
Adjuvant Treatment	aHR^*^	95% CI	*P* value	Adjuvant Treatment	aHR^*^	95% CI	*P* value	Adjuvant Treatment	aHR^*^	95% CI	*P* value
IV fluoropyrimidines	1.00			IV fluoropyrimidines	1.00			IV fluoropyrimidines	1.00		
Oral fluoropyrimidines	1.35	1.13-1.60	.0007	Oral fluoropyrimidines	1.29	0.62-2.71	.4932	Oral fluoropyrimidines	1.34	1.14-1.59	.0006

*All the aforementioned variables in Table 2 were used in the multivariate analysis. aHR, adjusted hazard ratio.

## Abbreviations

ICD-9-CM: International Classification of Diseases, Ninth Revision, Clinical Modification
AJCC: American Joint Committee on Cancer
HR: hazard ratio
CCI: Charlson comorbidity index
OS: overall survival
aHR: adjusted hazard ratio
TCRD: Taiwan Cancer Registry Database
PS: propensity score
IV: intravenous
5-FU: 5-fluorouracil
TP: thymidine phosphorylase
SMD: standardized mean difference
Cmax: maximum concentration
AUC: area under the curve
DPD: dihydropyrimidine dehydrogenase
CYP: cytochrome P
