# Supplementary material for: Outcomes of Adjuvant Oral versus Intravenous Fluoropyrimidines for High-Risk Stage II or Stage III Colon Adenocarcinoma: A Propensity Score-Matched, Nationwide, Population-Based Cohort Study
Source: J Cancer. 2020 Apr 12;11(14):4157–65. doi: 10.7150/jca.42404 (PMC7196259; doi:10.7150/jca.42404)
Supplement: Supplementary file 1 — Supplementary figures and tables. [file jcav11p4157s1.pdf]

Supplemental Figure 1. Kaplan–Meier curves for overall survival of patients with high-risk stage II or III colon cancer who received adjuvant oral or intravenous fluoropyrimidine

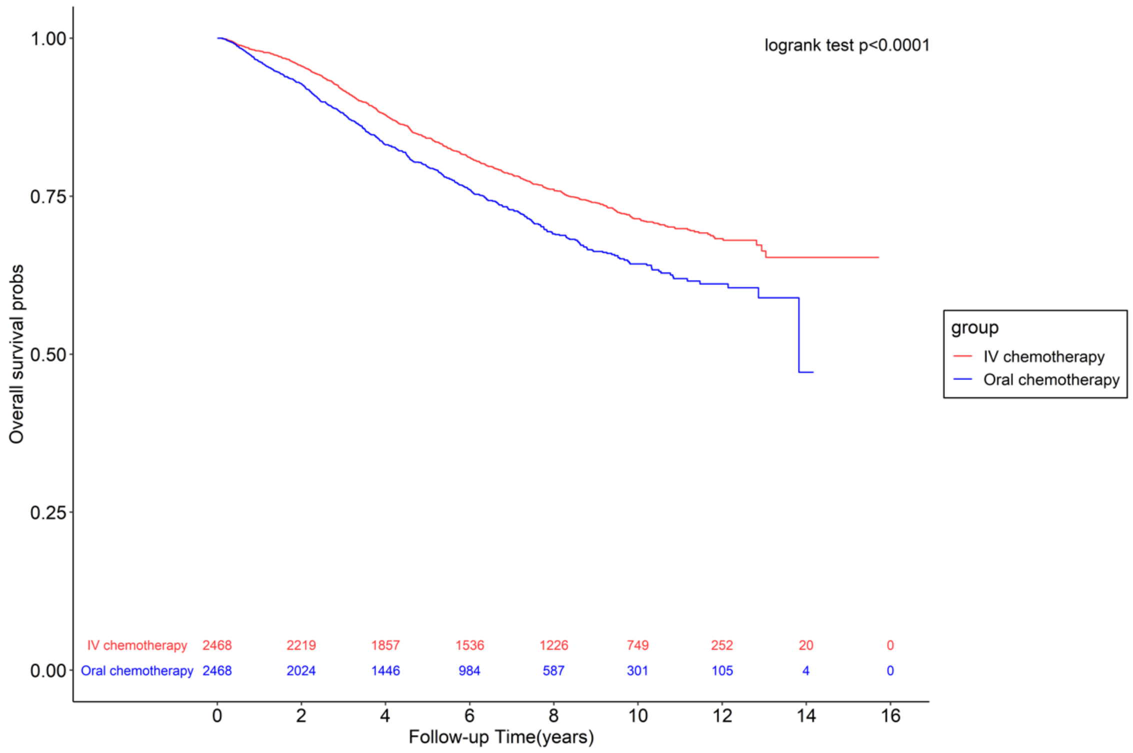

Supplemental Figure 2. Kaplan–Meier curves for overall survival of patients with high-risk stage II colon cancer who received adjuvant oral or intravenous fluoropyrimidine

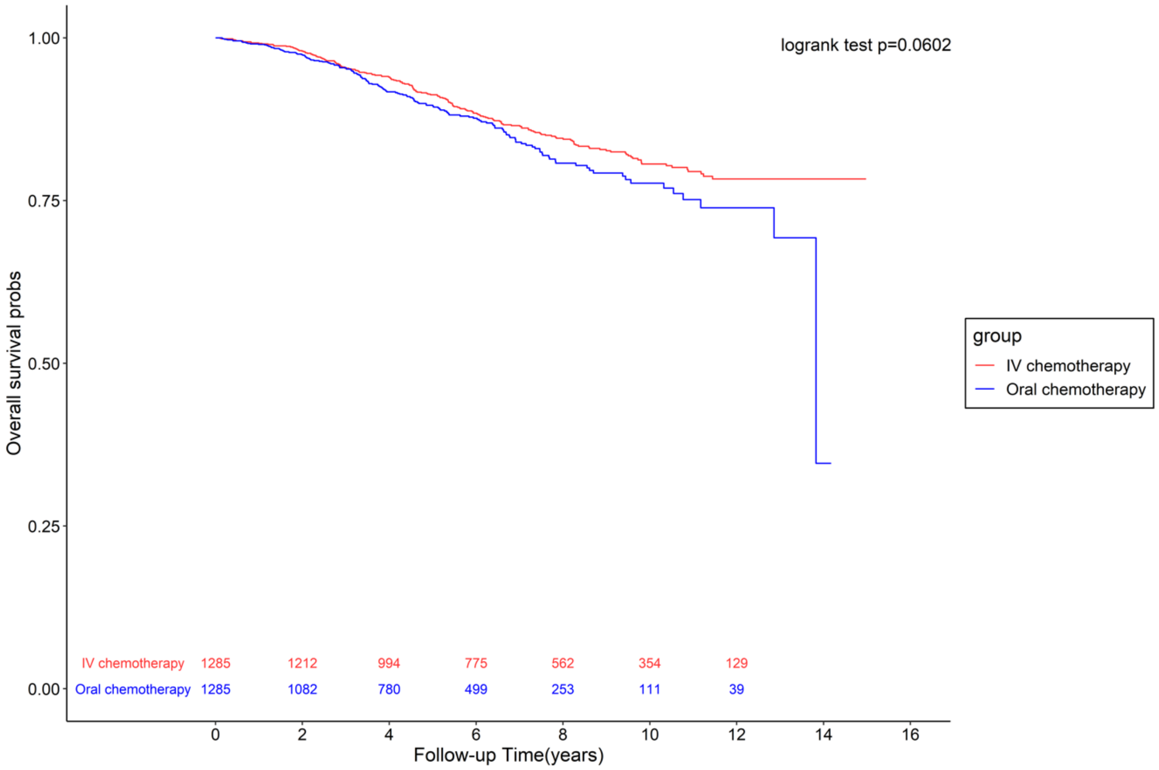

Supplemental Figure 3. Kaplan–Meier curves for overall survival of patients with stage III colon cancer who received adjuvant oral or intravenous fluoropyrimidine

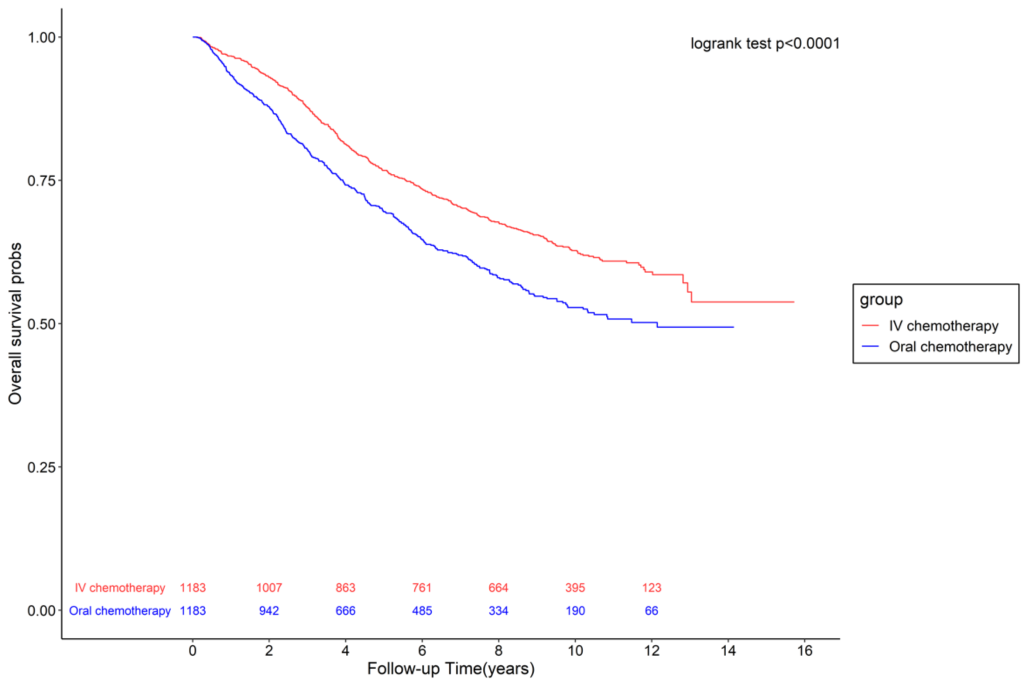

Supplemental Table 1. Characteristics of adjuvant oral or intravenous fluoropyrimidine chemotherapy for patients with colon adenocarcinoma who received curative surgery and their nonmatched cohort

	**Oral Fluoropyrimidine N = 2,468**	**IV Fluoropyrimidine N = 2,468**	**SMD**
**Adjuvant chemotherapy duration (days), median (IQR)**	286.5 (271.0)	201.0 (59.0)	-
**Follow-up time (years)**			
Mean (SD)	6.4 (3.4)	8.3 (3.7)	0.5623
Median (IQR)	5.8 (5.3)	8.1 (6.5)	-
**Total cycles of chemotherapy**			
Mean (SD)	5.4 (3.8)	4.8 (4.2)	0.3261
Median (IQR)	5.0 (3.0)	4.0 (4.0)	-
**Total cumulative dose of chemotherapy (mg/m^2)**			
Mean (SD)	109 411.5 (96 058.4)	5524.5 (2704.2)	1.5971
Median (IQR)	81 200.0 (95 200.0)	5700.0 (2800.0)	-

SMD, standardized mean difference; SD, standard deviation; IQR, interquartile range; IV, intravenous
